# The Green Anti-Cancer Weapon. The Role of Natural Compounds in Bladder Cancer Treatment

**DOI:** 10.3390/ijms22157787

**Published:** 2021-07-21

**Authors:** Paulina Wigner, Michal Bijak, Joanna Saluk-Bijak

**Affiliations:** 1 Department of General Biochemistry, Faculty of Biology and Environmental Protection, University of Lodz, Pomorska 141/143, 90-136 Lodz, Poland; paulina.wigner@biol.uni.lodz.pl; 2 Biohazard Prevention Centre, Faculty of Biology and Environmental Protection, University of Lodz, Pomorska 141/143, 90-136 Lodz, Poland; michal.bijak@biol.uni.lodz.pl

**Keywords:** bladder cancer treatment, curcumin, sulforaphane, resveratrol, quercetin, 6-gingerol, delphinidin, epigallocatechin-3-gallate, gossypol

## Abstract

Bladder cancer (BC) is the second most common genitourinary cancer. In 2018, 550,000 people in the world were diagnosed with BC, and the number of new cases continues to rise. BC is also characterized by high recurrence risk, despite therapies. Although in the last few years, the range of BC therapy has considerably widened, it is associated with severe side effects and the development of drug resistance, which is hampering treatment success. Thus, patients are increasingly choosing products of natural origin as an alternative or complementary therapeutic options. Therefore, in this article, we aim to elucidate, using the available literature, the role of natural substances such as curcumin, sulforaphane, resveratrol, quercetin, 6-gingerol, delphinidin, epigallocatechin-3-gallate and gossypol in the BC treatment. Numerous clinical and preclinical studies point to their role in the modulation of the signaling pathways, such as cell proliferation, cell survival, apoptosis and cell death.

## 1. Introduction

Bladder cancer (BC) is the second most common cancer of the genitourinary system. In 2018, BC was diagnosed in 550,000 people worldwide, while nearly 200,000 BC patients died. Interestingly, despite significant advances in medicine over the past 15 years, the death rate related to bladder cancer has only decreased by 5%, while the death rates related to prostate, breast, lung and colon cancer have fallen more sharply (a drop by up to a dozen or so percent) [1]. The regions with the highest incidence of BC include Southern and Western Europe and North America. Moreover, men suffer from BC four times more often than women. This higher risk of BC development in men is probably associated with a higher percentage of smokers among men than women. Previous epidemiological studies have confirmed that cigarette smoking increases the risk of BC development. This fact also confirms the elevated incidence of BC among women in the developed world, which is characterized by an increase in the percentage of smokers among women [2]. Moreover, a total of 90% of BC diagnoses are made in those 55 years of age and older, and 7 out of every 10 cases of BC are detected in the early stages [1,2]. Although BC is diagnosed as a superficial disease when detected early, it is characterized by its high recurrence risk. The competing-risks regression analysis showed that the 2-year, 5-year and 10-year BC recurrence rates were 61.1%, 69.5% and 74.3%, respectively [1]. Moreover, further disease progression to a muscle-invasive stage is associated with a worse prognosis in patients after recurrence. The progression of BC is associated primarily with the formation of unpredictable metastases, which is a consequence of the increased invasiveness of cancer cells. The appearance of metastases complicates the course of the disease and its treatment, which in turn results in a high mortality rate among BC patients. Thus, BC treatment is a major challenge in modern medicine [3,4,5]. Standard treatment of bladder cancer depends on many factors, such as the type, grade and stage of cancer, which are taken into consideration along with one’s overall health and treatment preferences. Conventional BC treatment may include: (i) surgery, (ii) intravesical chemotherapy, (iii) systemic chemotherapy, (iv) radiation therapy, (v) immunotherapy and/or (vi) targeted therapy.

TURBT (transurethral resection of bladder tumour) is the primary method of diagnosis and treatment of non-muscle-invasive bladder cancer (TaT1) and should be performed systematically in individual steps. Adjuvant treatment for all stages (TaT1 and CIS-carcinoma in situ) is the administration of intravesical drugs. The choice of the type of therapy depends on the risk for recurrence and/or progression of tumors after TURBT. The types of intravesical therapy are as follows: immediate single postoperative instillation of chemotherapy (mitomycin C, epirubicin, doxorubicin); further chemotherapy and intravesical immunotherapy with BCG (Bacillus Calmette-Guerin, which is superior to intravesical chemotherapy in reducing recurrences and in preventing or delaying progression to muscle-invasive bladder cancer). In turn, for muscle-invasive bladder cancer (MIBC) and non-muscle invasive bladder cancer (NIMBC) at the highest risk of progression to MIBC, the primary treatment modality is radical cystectomy to remove the entire bladder and surrounding lymph nodes. In patients with bladder cancer in the muscular infiltrating stage cT2-T4aN0M0, the currently recommended therapy is radical cystectomy preceded by cisplatin-based neoadjuvant chemotherapy. It is possible to perform a partial cystectomy, but these are selected patients with a properly functioning bladder with good capacity and a single localized tumor [6,7].

External beam radiotherapy alone should only be considered as a therapeutic option when a patient is unfit for cystectomy or as a part of the trimodal bladder-preserving approach (TMT). Moreover, radiotherapy can also be used to stop bleeding from the tumor when local control cannot be achieved by transurethral manipulation due to extensive tumor growth [6].

In addition to intravesical therapy used after TURBT intravesical chemotherapy, one of the methods of antitumor treatment is systemic chemotherapy consisting of administering drugs in the form of tablets or injecting into a vein or muscle [6]. Chemotherapy can also be given in conjunction with radiation; in some cases, alternative combination therapy is used to increase the effectiveness of anticancer treatment consisting of maximal transurethral resection followed by concurrent chemoradiotherapy (also known as trimodal therapy, TMT). TMT is an alternative to selected well-informed and compliant patients, especially for whom radical cystectomy is not an option or not acceptable [6,8]. For metastatic bladder cancer, first-line chemotherapy is cisplatin-combination chemotherapy (GC or HD-MVAC) for platinum-fit patients. In patients unfit for cisplatin but fit for carboplatin, we use a combination of carboplatin with gemcitabine. The second-line treatment for metastatic disease is immunotherapy as checkpoint inhibitor pembrolizumab. If this is not possible, atezolizumab, nivolumab (European Medicines Agency (EMA), Food and Drug Administration (FDA) approved), avelumab or durvalumab (FDA approved). Further treatment after platinum and immunotherapy are in clinical trial testing novel antibody–drug conjugates or, in the case of patients with FGFR3 (fibroblast growth factor receptor 3) alterations, FGFR tyrosine kinase inhibitors [6,7,8]. However, due to the lack of alternative therapies, some drugs receive accelerated approval (enfortumab vedotin, sacituzumab govitecan) for some BC patients. The accelerated approval is dedicated to accelerating the development and registration process of a promising medicinal product, assessed in research in terms of future use in the treatment of patients in severe and threatening the life of clinical states while guaranteeing a high level of patient safety. It is used to be used for products showing a potential clinical advantage towards severe patients for which there is no appropriate therapeutic alternative. In turn, the full registration of the FDA means that the drug has passed all the required quality, efficacy and safety tests, and thus has been registered and admitted to trading [9]. In the case of enformaband, in 2019, it has been published accelerated approval; however, after further studies, this drug received full FDA approval in 2021. The full FDA approval of enformaband means that the drug has been officially approved and approved for the treatment of patients who: (i) received a programmed death receptor-1 (PD-1) or programmed death-ligand (PD-L1) inhibitor and chemotherapy containing platinum, (ii) are not eligible for chemotherapy containing cisplatin and received at least one treatment line earlier [10]. In 2021, the FDA granted accelerated approval to sacituzumab govitecan (Trodelvy) for the treatment of patients with locally advanced or metastatic urothelial cancer (mUC) who previously received platinum-containing chemotherapy and either a PD-1 or PD-L1 inhibitor. This approval was based on the final data from cohort 1 of the phase 2 TROPHY-U-01 trial, which showed that sacituzumab govitecan induced an overall response rate (ORR) of 27% in heavily pre-treated patients with mUC following the failure of both platinum-based chemotherapy and checkpoint inhibition. In the case of safety, diarrhea was the most commonly experienced treatment-related adverse effect, occurring in two-thirds of patients; however, most of these effects were grade 1/2 [9].

However, despite the development of techniques of BC treatment in the last decades, the mean survival time of a patient with high-grade malignancy after relapse has been estimated as merely 5.6 months [11]. Moreover, BC therapy is also associated with severe side effects and the development of drug resistance that hamper treatment’s success. Thus, high-risk patients are currently given several intravenous therapeutic agents to inhibit disease relapse and progression. Unfortunately, the combination of different therapeutic agents is also associated with very high costs [12,13]. In consequence, patients are increasingly choosing cheaper products of natural origin as an alternative or complementary therapeutic options. In this article, we aim to elucidate, using the available literature, the role of natural compounds, including curcumin, sulforaphane, resveratrol, quercetin, 6-gingerol, delphinidin, epigallocatechin-3-gallate and gossypol, in BC treatment.

## 2. Therapeutic Potential of Curcumin in Bladder Cancer

**Figure 1:**
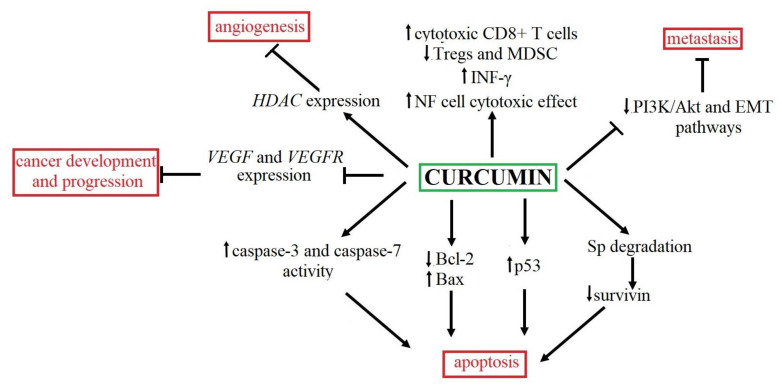
The summary of anticancer effects and mechanisms of curcumin action. Curcumin may modulate numerous signaling pathways, including angiogenesis [16], immune response [25,26,27], cancer development and progression [15,21,22,23] as well as apoptosis [15,17,18,19,20].

One of the complementary BC therapeutic agents may be curcumin, characterized by chemopreventive effects in experimental cancer models. Curcumin (diferuloylmethane) is a major, safe and non-toxic compound of the spice turmeric derived from the rhizomes of *Curcuma longa*, which is characterized by demonstrable antitumor, anti-inflammatory, antiapoptotic and antioxidant properties [14]. Moreover, the previous results confirmed that curcumin might modulate numerous signaling pathways, such as cell proliferation, cell survival, apoptosis and cell death, displaying a high potential for anticancer therapy [15]. Additionally, this compound plays a crucial role in protecting organs from toxicity induced by chemotherapy. Recently, it has been confirmed that curcumin showed the histone deacetylase (HDAC) inhibitor potential. Histone modification is a crucial mechanism in cancer development and progression. The study on healthy human volunteers showed that oral curcumin administration reduced *HDAC* expression without side effects compared to synthetic HDAC inhibitors using clinical practice [16]. Anticancerous properties of curcumin, also associated with apoptosis induction, mainly involve mitochondria. Liu and colleagues (2011) found that curcumin treatment led to an increase of p53 level while down-regulating the antiapoptotic protein Bcl-2 (B-cell lymphoma 2) [17]. Moreover, the rat bladder carcinogenesis model showed that the curcumin treatment was associated with the increased expression of the pro-apoptotic Bcl-2 associated X protein (*Bax*) [18]. Animal and in vitro studies showed that curcumin induces the elevated activity of executive caspases, such as caspases-3 and 7 [18,19]. Additionally, curcumin application caused a decreased level of survivin in BC cell lines. Survivin belongs to the inhibitor of apoptosis (IAP) protein family, preventing apoptosis by reducing caspases-3, -7 and -9 activity [18,20]. The reduced survivin level following curcumin application may be associated with the degradation of the specificity protein (Sp). Besides the survivin level modulation, Sp-proteins are involved in decreased vascular endothelial growth factor (*VEGF*) and VEGF receptor 1 (*VEGFR1*) expression. Thus, curcumin also showed antiangiogenic potential [21]. Adhesion-blocking curcumin properties (inhibition of tumor implantation) were first noticed 20 years ago in a murine bladder tumor model [22]. It has been suggested that the anti-invasion and antimigration character of curcumin may result from modulation of PI3K/AKT (phosphatidylinositol 3-kinase/protein kinase B) and EMT (epithelial–mesenchymal transition) pathways [23]. Curcumin may also block integrin adhesion receptors, thereby limiting migration and invasion of BC cells and slowing down the formation of BC metastasis [24]. Anticancer properties of curcumin may also be associated with regulating adaptive and innate immunity. As part of adaptative immunity, curcumin increases the level of cytotoxic CD8+ T-cells, decreases Tregs and myeloid-derived suppressor cell (MDSC) levels [25] and enhances interferon-gamma production [26]. On the other hand, innate immune modulation enhances the cytotoxic effect of NK (natural killer) cells [27]. The summary of the antitumor activity of curcumin is presented in Figure 1.

Moreover, curcumin could also be combined with conventional therapy. It can support BCG therapy through potentiated BCG-induced apoptosis of human BC cells. Previous studies showed that BCG induced the release of tumor necrosis factor-related, apoptosis-inducing ligand (TRAIL) from peripheral mononuclear neutrophils, while curcumin increased the TRAIL receptors upregulation. An in vitro study showed that curcumin might intensify BCG-induced activation of caspase-8 and caspase-9. The clinical potential of BCG therapy supplemented with curcumin had also been confirmed by the fact that this combined therapy suppressed NF-κB (nuclear factor kappa-light-chain-enhancer of activated B cells) activation. This pathway is crucial for certain tumor cells to escape apoptosis. The decreased NF-κB activity led to downregulated NF-κB–regulated cancer cell survival proteins, such as Bcl-2, Bcl-xL (B-cell lymphoma-extra large) and survivin, both in vitro and in vivo [28]. Another mechanism associated with the anticancer properties of curcumin is its interaction with miRNA. Curcumin caused a downregulation of the tumor-promoting miR-7641, which increased *p16* tumor suppressor gene expression. Finally, p16 tumor suppressor gene caused apoptosis induction [29].

Curcumin could also impact immunotherapy effectiveness. In vivo study confirmed that the curcumin administration might lead to induction of tumor antigen-specific T cells in the restoration of dendritic cells pathway directly by inhibiting STAT3 (signal transducer and activator of transcription 3) and indirectly via reduced IL-6 (interleukin 6) production from STAT3 activated cancer cells in the murine tumor models. STAT3 contributes to immunosuppression in the tumor microenvironment by the induction of immunosuppressive cytokines production in cancer cells, including IL-6, IL-8 and VEGF. Moreover, obtained results showed that STAT3 depletion in dendritic cells led to the enhancement of their function and subsequent T cell induction. Thus, STAT3 may be a potential therapeutic target in BC. Hayakawa et al. (2020) found that curcumin could augment antitumor T cell responses by inhibiting STAT3 activated cancer cells and dendritic cells as well as showed synergistic antitumor effect with anti-PD-1/PD-L1 antibodies leading to enhance anticancer immune responses and induction of tumor cell death [30]. PD-1 is expressed on activated T cells, B cells, monocytes, dendritic cells, regulatory T cells and natural killer T cells as well as tumor-infiltrating lymphocytes (TILs), while tumor cells are commonly characterized by upregulated *PD-L1* as compared to normal cells. The receptor of PD-L1 is PD-1. Under normal conditions, the PD-L1/PD-1 connection determines the maintenance of the peripheral immune tolerance and protects against excessive tissue inflammation and autoimmune disease. In turn, in the course of the cancer, the combination of PD-1 and PD-L1 inhibits the antitumor immunity, resulting in a tumor immune escape on the way of (i) inhibition of TILs activation and induced their apoptosis, (ii) reduction of the secretion of the inflammatory cytokines, including IFN-γ (interferon γ), IL-2, TNF-α (tumor necrosis factor α) and induced immune inhibitory cytokine secretion, such as IL-10, IL-4) stagnating the T cell cycle. As a consequence, these processes lead to the promotion of the tumor cell epithelial materialization, metastasis and infiltration formation [31].

Previous studies also showed that resistance to anticancer treatment could be eliminated by the use of curcumin. Gemcitabine resistance of BC cells can be reversed by simultaneous treatment with curcumin. The combined treatment caused an additive cytotoxic effect and reduction of the tumor migration [32]. On the molecular level, curcumin intensified the apoptotic action of gemcitabine by upregulating TRAIL and modulating the NF-κB pathway. Additionally, curcumin caused the suppression of genes associated with proliferation and angiogenesis, including cyclooxygenase-2 (COX-2) and VEGF [26]. An animal study showed that cisplatin treatment combined with curcumin reduced the size of the tumor after 27 days, while no response was observed when curcumin or cisplatin was applied alone [33]. The molecular mechanism of cisplatin and curcumin combined therapy includes two pathways: (i) curcumin may potentiate cisplatin-induced apoptosis via reactive oxygen species (ROS)-mediated activation of ERK1/2 (extracellular signal-regulated kinase 1/2) or (ii) combined therapy may induce upregulating pro-apoptotic *Bax* and down-regulating antiapoptotic *Bcl-2* and the X-linked inhibitor of apoptosis protein (*XIAP*) in the dependent or independent p53 pathway [33].

Interestingly, curcumin may protect organs from chemotherapy-induced toxicity. Cisplatin therapy is associated with severe adverse effects, including acute kidney damage, affecting up to 60% of patients [34]. Mice study showed that curcumin decreased cisplatin-induced nephrotoxicity by reducing serum and renal TNF-α and renal monocyte chemoattractant protein (MCP)-1 concentrations [35]. Moreover, curcumin may also attenuate mitochondrial oxidative damage in kidneys observed after cisplatin therapy [36]. Curcumin itself in very high doses (over 2000 mg per day) shows an adverse effect, but they are very weak and involves neutropenia, diarrhea, abdominal swelling or pain, transient red blood cell echinocyte formation and an increase in the mean red blood cellular volume [37,38,39,40].

## 3. Therapeutic Potential of Broccoli Sulforaphane in BC

For the first time, the anticancer properties of broccoli were confirmed in 1997 by Fahey’s team [41]. Two years later, the large prospective cohort study showed that ≥2 servings of broccoli/week caused a 39% reduction in risk compared with <1 serving [42]. The unusual properties of broccoli are related to its ingredients. Broccoli contains carotenoids, vitamin C, glucosinates and sulforaphane (SFN), which is derived from a glucoraphanin (GPN) precursor [43]. GPN conversion into SFN occurs while cutting or chewing broccoli, which releases the endogenous heat-labile myrosinase enzyme [44]. The enzyme is destroyed during cooking or even steaming or blanching for more than a minute. However, mild steaming and microwaving may retain more SFN than conventional boiling [45,46]. Moreover, GPN is several times more prevalent in sprouts than whole mature heads [44].

Rat study showed that dietary administration of freeze-dried aqueous extract of broccoli sprouts significantly and dose-dependently inhibited BC development induced by N-butyl-N-(4-hydroxybutyl) nitrosamine, including lower cancer incidence, smaller tumor size and reduced cancer invasiveness. Bladder cells carcinogenesis inhibition by the broccoli extract was associated with increased induction of glutathione S-transferase (GST) and NAD(P)H: quinone oxidoreductase 1 (NQO1), which are important protectant enzymes against oxidants and carcinogens. GSTs are crucial phase II enzymes that generally detoxify carcinogenic metabolites involving the conjugate of many electrophilic substrates with glutathione. Therefore, this enzyme family protects from chemical-induced cancer, including polycyclic aromatic hydrocarbon epoxides and acrylamides. Thus, the correlation between the induction of GST and NQO1 by the broccoli extract in the bladder and inhibition of bladder carcinogenesis suggest that induction of these enzymes may act as a reliable biomarker in the inhibition of bladder carcinogenesis by broccoli [47,48]. However, due to the administration of SFN before and during exposure to carcinogens, the broccoli’s anticancerous effect is characteristic only for the tumor initiation phase [48]. Subsequent polymorphism analysis also showed that *GSTM1* null genotype was associated with increased BC risk in the Turkish population, which further increased in smokers [49]. Similarly, polymorphism localized in the *NQO1* gene was associated with the BC development risk. A previous study showed that a C-->T single nucleotide polymorphism in exon 6 was shown to reduce NQO1 enzyme activity. Thus, the C/T and T/T genotypes of the SNP were associated with an increased risk of BC development in Caucasians, especially in the group of smokers [50].

Moreover, the in vitro studies showed that isothiocyanate extract of broccoli sprouts induced mitochondria-mediated apoptosis and also halted cells in S and M phases. The apoptosis activation includes mitochondrial damage, cleavage of caspase-3/9 and poly (ADP-ribose) polymerase (PARP), cytoplasmic accumulation of histone-associated DNA fragments and absence of cleavage of caspase-8. Cell cycle arrest (in S and M phases) resulted from the down-regulation of Cdc25C and mitotic spindles disruption [51]. Moreover, the anticancer effects of SFN (mitotic arrest and apoptosis) are regulated via reactive oxygen species (ROS)-dependent mechanisms [52]. In addition, apoptosis of BC cells after SFN treatment is activated by dysregulation of mitochondria function pathway, including cytochrome c release and Bcl-2-related pathways [53]. Additionally, SFN may modulate the expression of genes involved in apoptosis and proliferation via epigenetic changes. Thus, SFN and broccoli sprouts are considered as an “epigenetics diet” and may inhibit HDAC activity and inhibition of DNA methyltransferases (*DNMT*) expression [54]. HDAC is a crucial enzyme of histone acetylation and thus it modulates transcription factor access to DNA, while DNMT affects gene expression [54].

On the other hand, SFN may modulate angiogenesis. In vitro study showed that SNF inhibited the hypoxia-induced mRNA expression of *VEGF*, hypoxia-inducible factor-1α (*HIF-1α*) and *c-Myc* in an immortalized human microvascular endothelial cell line, HMEC-1. Moreover, a 3-week sulforaphane treatment of placental vessel fragments inhibited angiogenic capillary growth. Angiogenesis limit may result from the inhibition of NF-κB-mediated expression of inducible nitric oxide synthetase (*iNOS*) and *COX-2*, which contribute to *VEGF* expression. Additionally, SFN can also impact membrane integrity, and it suppresses transcription of the predominant endothelial collagenase matrix metalloproteinase-2 and its tissue inhibitor of metalloproteinase-2 [55].

SFN may also protect the normal bladder cell against chemical-induced DNA damage. One of the toxic compounds is 4-aminobiphenyl (ABP), contained in tobacco smoke, which increases BC risk. Both human bladder cells in vitro and mouse bladder tissue in vivo studies confirmed that SFN inhibits ABP-induced DNA damage via activation of NF-E2 related factor-2 (Nrf2). Nrf2 transcriptionally activates many cytoprotective genes, including carcinogen-detoxifying phase II genes, which play a pivotal role in the cellular defense against various chemical carcinogens, including derivatives of tobacco smoke [56,57].

An additional advantage of broccoli extracts is their relative selectivity. Studies on the metabolism of broccoli isothiocyanates, including SFN, showed that urinary isothiocyanate concentrations were 2–3 orders of magnitude higher than in plasma within 24 h after dosing. Moreover, isothiocyanate levels in the bladder were 2.7 to 19.7 times higher than in the liver, compared to the same tissue weight. Therefore, the obtained results indicate that isothiocyanates are selectively delivered to the bladder and confirm that the test compounds may be particularly useful in the prophylaxis of bladder cancer [48]. Notably, the obtained results confirmed that SFN is selectively more toxic to malignant urothelial cells (human) than normal urothelium [58]. Moreover, combined therapy of acetazolamide with SFN marked higher antitumor efficacy by inhibition of PI3K/Akt/mTOR pathway than when the studied compound was applied alone [59]. In addition to selectivity, SFN has high bio-availability (74%) and is also quickly excreted in the urine, thus increasing the elimination of harmful carcinogens [60].

In primary human T cells, SFN also acts pro-oxidatively, increasing intracellular ROS levels and decreasing GSH (glutathione). In consequence, SFN can inhibit T cell activation and T cell effector functions. Thus, there are two sides to SFN in cancer treatment. On the one hand, the compound may reduce carcinogenesis, but on the other, it blocks the T cell-mediated immune response. Therefore, SFN could also interfere with the successful immunotherapy (e.g., CTLA-4-blocking antibodies and PD-1/PD-L1-blocking antibodies), and the SFN combination therapy with T cell-mediated immunotherapies does not seem advisable [61].

**Figure 2:**
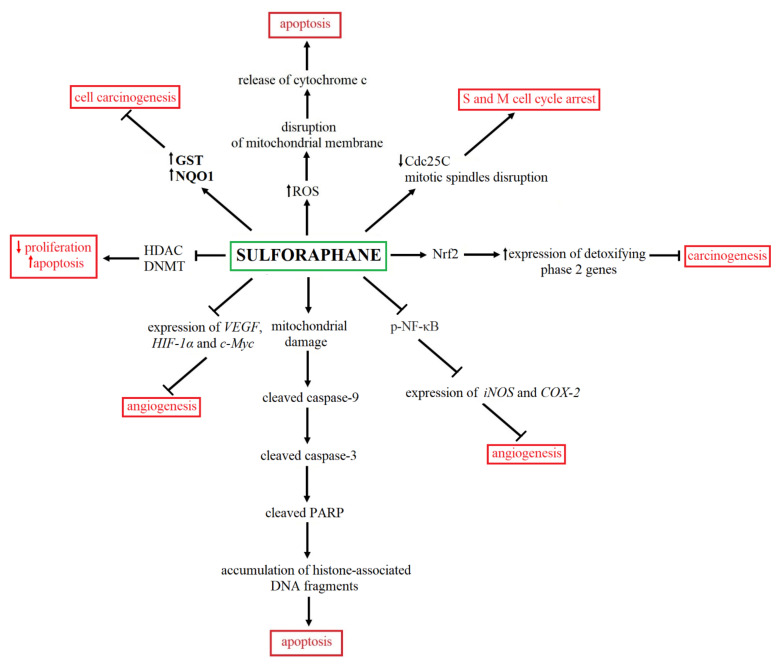
The summary of anticancer effects and mechanism of SFN action. SFN may modulate numerous signaling pathways, including angiogenesis [55], cell carcinogenesis [47,48,49,50,56,57], cell proliferation [54] and cell cycle [51,52] as well as apoptosis [51,52,53,54].

Moreover, isothiocyanates, including SFN, may also be used in BC prevention. Michaud et al. (2007) confirmed the protective effect of isothiocyanates on bladder carcinogenesis in a male cohort. Cruciferous vegetable intake twice decreased bladder cancer risk [40]. Unfortunately, in the case of many cancers, previous studies showed that genetic and associated functional variations in various genes, including these encoding biotransformation enzymes, may lead to individual differences in cancer risk modulation in response to cruciferous vegetable intake [62,63]. However, in the case of BC, Zhao and colleagues (2007) found that *NAT2* (N-acetyltransferase 2) slow genotype increased about 37% BC risk in the Caucasian population but did not impact the isothiocyanate protective anticancer effect [64]. The summary of the antitumor activity of SFN is presented in Figure 2.

## 4. Therapeutic Potential of Resveratrol in BC

**Figure 3:**
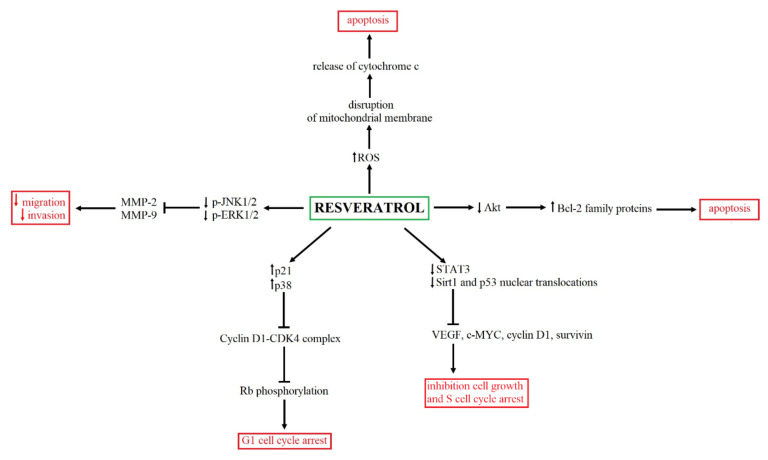
The summary of anticancer effects and mechanism of RSV action. RSV may modulate numerous signaling pathways, including cell cycle [73,74,75,76,80,81,82], metastasis [83], as well as apoptosis [77,78,79].

Resveratrol (RSV) is a polyphenolic compound, which occurs in grapes, blackberries, blueberries, raspberries and peanuts [65]. Previous studies showed that RSV has a wide range of health-promoting properties, including cardio-protection, antioxidant, anti-inflammatory, antibacterial and antifungal, antiaging, neuroprotective as well as anticancer [66,67,68,69,70,71,72]. Numerous in vitro studies have confirmed that RSV led to G1 cell cycle arrest in transitional cell carcinoma, causing p21 and p38 activation [73,74]. In turn, the increased expression of *p21* and *p38* may lead to Cyclin D1-CDK4 complex inhibition, which is the mediator of G1-S transition and inhibits Rb phosphorylation [75,76]. Moreover, the cell cycle arrest may be accompanied by apoptosis, induced through inhibition of the p-Akt signaling pathway, contributing to cancer progression by promotion of cell proliferation and apoptosis suppression [77]. p-Akt inhibition may lead to apoptosis as a result of mitochondrial pathways activation and modulation of Bcl-2 family protein activity [77,78]. In vitro studies confirmed that RSV exposition disrupted a mitochondrial membrane potential, resulting in cytochrome c release. In the cytosol, released cytochrome c binds to Apaf-1, which recruits and activates caspase-9 and further leads to the activation of effector caspases and, in consequence, causes cell death [79]. Interestingly, the subsequent study showed that RSV-exposed transitional carcinoma cells were characterized by reduced cell growth, apoptosis and cell cycle arrest, which were accompanied by inhibition of the STAT3 (signal transducer and activator of transcription) signaling pathway, Sirt1 (Sirtuin 1) and p53 nuclear translocations [80]. STAT3 modulates the expression of genes involved in tumor development and progression, including *VEGF*, *c-Myc*, *cyclin D1* and *survivin* [81]. In turn, Sirt1 impacts cell death and survival by deacetylating transcriptional factors, including p53 [82]. RSV-induced apoptosis may be a consequence of the influence of RSV on miR-21, which is known as an oncogene, enhancing cell proliferation, promoting cell cycle progression and increasing antiapoptotic activation in cancer cells. A previous study showed that miR-21 upregulation was characterized for advanced bladder cancer tissues [83]. The next in vitro study showed that RSV inhibits miR-21 expression, causing the reduction of Akt activity through reduced phosphorylation, which results in a decrease in *Bcl-2* expression. However, miR-21 overexpression reversed the effect of resveratrol on BC cells [84]. The summary of the antitumor activity of RSV is presented in Figure 3.

RSV treatment may also impact cancer metastasis. In vitro RSV reduced JNK1/2 (c-Jun N-terminal kinases 1/2) and ERK1/2 phosphorylation, causing the inhibition of MMP-2 (matrix metalloproteinase-2) and MMP-9 (matrix metalloproteinase-9) activity, which are involved in cell migration and invasion [85,86,87]. Similarly, in vivo studies confirmed that RSV treatment slowed the growth of tumors resulting in reduced expression of the proangiogenic factors, such as *VEGF* and *FGF-2* (Fibroblast growth factors 2). In turn, Wu and colleagues (2014) confirmed the tumor growth inhibition after RSV intravesical treatment due to STAT3 signaling pathway inhibition (reduced expression of *survivin*, *cyclin D1*, *c-Myc* and *VEGF*) [80].

RSV and piceatannol act synergistically and may also be involved in the induction of *PD-L1* expression. In cancer cells, disorders of *PD-L1* expression and its binding to PD-1 of cytotoxic T cells act as a “stop sign” and counter the proliferation and function of T cells, thus augmenting the tumor evasion of host antitumor immunity. Thus, RSV- piceatannol-induced *PD-L1* expression may serve as a Search, Enhance and Engage (“SEE”) signal to sensitize low *PD-L1*-expressing “non-responsive” tumors, augmenting their detection by PD-1/PD-L1 immune checkpoint blockade (PD-1 and PD-L1 inhibitors) and finally leads to the elimination of cancer cells by antitumor immunity. However, it should be emphasized that RSV can only be used in combination with anti-PD-1/PD-L1 antibodies as it alone promotes tumor progression [88]. Moreover, RSV may be used in combination with conventional BC treatment. RSV and rapamycin (inhibitor of rpS6 kinase) combined therapy effectively inhibit mTOR regulation (effectors such as p-56K1, p-S6, p-4EBP1 and p-eIF4B), which is crucial for cell growth, proliferation and survival. Thus, this therapy was also associated with apoptosis, reduced cell migration and clonogenic survival of BC cells [89]. Additionally, RSV may also contribute to the sensitization of drug-resistant cancer cells. Cho et al. (2019, 2020) showed that RSV combination with gemcitabine leads to an additive cytotoxic effect in gemcitabine-resistant BC cells. Although the counter-resistance mechanism remained unclear, it involved factors other than ATP binding cassette subfamily C member 2 (ABCC2), deoxycytidine kinase (DCK), thymidine kinase 1 (TK1) and thymidine kinase 2 (TK2). Unfortunately, the future use of RSV is limited due to its low bioavailability and fast metabolism [32,90]. Thus, further studies should focus on developing RSV transport systems to the destination [91,92,93].

## 5. Therapeutic Potential of Quercetin in BC

Quercetin (3,3′,4′,5,7-pentahydroxyflavone) is the natural polyphenolic flavonoid compound, occurring mainly in citrus fruits, apples, onions, parsley, tea and red wine are the primary dietary sources of quercetin. Additionally, olive oil, grapes, dark cherries and dark berries (blueberries, blackberries and bilberry are characterized by a high concentration of flavonoids, including quercetin) [94].

In vitro study showed that quercetin treatment caused suppression of the BC cells’ proliferation or motility, which resulted in increased expression of p-AMPK and decreased p-p70s6k and p-4EBP1 [95]. Quercetin-induced proliferation inhibition may also be associated with Ca2+-activated K (KCa) channels. Quercetin-evoked hyperpolarisation caused K+ efflux through KCa activation and may eventually trigger apoptosis or inhibition of tumor cell proliferation, at least in bladder cancer cells [96].

In vitro, quercetin increased the caspase 3/7 activities, percentage of subG0/G1 cells and DNA fragmentation, associated with induced apoptosis. However, quercetin-decreased cell viability may be a result of autophagy. Human BC cells after quercetin exposition were characterized by the increased level of a specific marker of autophagy, such as LC3-II. On the other hand, combined treatment with autophagy inhibitor (bafilomycin A1) and quercetin may more effectively reduce bladder cancer cells’ proliferation through enhanced apoptosis [97]. In vivo study showed that quercetin might induce apoptosis of BC cells by regulating p53. Ma et al. (2006) showed that expression of mutP53 protein (mutant P53) decreased due to treatment with quercetin. Moreover, this treatment was also associated with the reduced expression of survivin [98]. The antiproliferative effect of quercetin on bladder transitional cell carcinoma may also be associated with, at least in part, disorders of the extracellular catabolism of nucleotides, which may be the result of AMP accumulation or quercetin-blocked adenosine receptors. Ecto-5′-nucleotidase (the main enzyme of nucleotide catabolism), catalyzing AMP hydrolysis into adenosine, has been described as a crucial compound involved in cancer progression, control of cell growth, maturation, differentiation, drug resistance and tumor-promotion. In vivo study confirmed that quercetin increased ADP hydrolysis and inhibited the ecto-5′-nucleotidase/CD73 activity, with no effect on protein expression, and finally caused the cell proliferation reduction [99]. Additionally, Oršolić and colleagues (2016) showed that in vitro quercetin contributed to the elevated level of DNA damage, including single- and double-strand breaks and alkali-labile sites [100]. Quercetin-induced DNA damage may be a consequence of a loss of mitochondrial transmembrane potential and induction of ROS overproduction, which finally may also lead to apoptosis [100].

**Figure 4:**
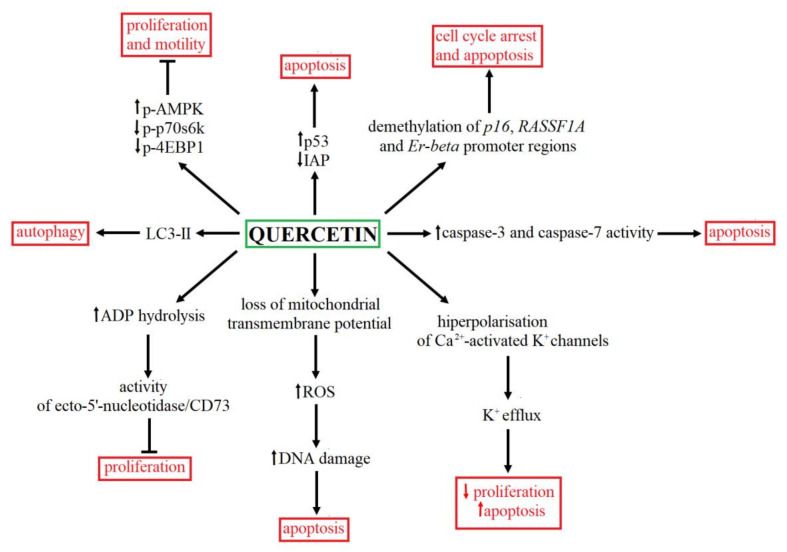
The summary of anticancer effects and mechanism of quercetin action. Quercetin may modulate numerous signaling pathways, including cell cycle [98] and proliferation [95,96,99], autophagy [97] as well as apoptosis [96,97,98,99,100].

Another mechanism of action of quercetin is associated with modulation of the methylation status of promoter regions of genes involved in tumor suppression through the cell cycle control (P16INK4a or CDKN2A and RASSF1a for Ras associated domain family protein 1 isoform A) and estrogenic transduction (estrogen receptor beta Er-beta or ESR2). The demethylation of *p16*, *RASSF1A* and *Er-beta* in BC cells led to restoring these genes’ transcriptional activity, resulting in death-associated protein synthesis and finally cell growth arrest and apoptosis of cells after quercetin treatment [98]. The summary of the antitumor activity of quercetin is presented in Figure 4.

Similar to SFN, quercetin can also be preventive in the development of BC. Sacerdote et al. (2007) showed that the consumption of cruciferous and green vegetables, which are the main source of quercitin, delphinidin (DPN), epigallocatechin-3-gallate (EGCG), would reduce the risk of developing BC both in the general population and in the group of smokers [101]. In addition, the obtained results confirmed no impact of polymorphisms in genes involved in DNA repair mechanisms (XRCC1-28152 (Arg399Gln, 28152G>A), XPD-35931 (Lys751Gln, 35931 A>C) and XRCC3-18067 (Thr241Met, 18067 C>T)) on the anticancer protect potential of cruciferous and green vegetable consumption [101].

Due to the low absorption, extensive metabolism and rapid elimination of natural quercetin, researches on its synthetic derivatives are currently being developed [102]. One of them is synthetic quercetin–zinc complex (Q-ZnCPX). Q-ZnCPX effectively inhibited movement and invasiveness of human bladder transitional carcinoma cells through p-Akt and membrane type-1 matrix metalloproteinase (MT1-MMP) pathway regulations. Q-ZnCPX could markedly down-regulate p-Akt/Akt protein expressions and result in decreased induction of MT1-MMP expression [103]. Another synthetic complex with quercetin includes quercetin in sodium (NaTNTQc) and zinc (ZnTNTQc) titanate nanotubes. In vivo study showed that maximum quercetin delivery was observed after 24 h, and its release was controlled by Zn. Moreover, both NaTNTQc and ZnTNTQc nanostructures decreased the viability of BC cells after 48 h of exposure [104].

Interestingly, the therapeutic potential of quercetin has also been confirmed by research on combination therapy. Cyclophosphamide is a nitrogen mustard alkylating agent, which can be administered in combination with paclitaxel in urothelial BC. However, there are no data about the effectiveness of cyclophosphamide alone administered. Lorenzo’s team (2016) was the first to describe a clinical case of a BC patient suffering from grade 2 fatigue, to whom there was the administration of a third-line, single-agent metronomic oral cyclophosphamide plus oral doses of quercetin [105]. The patient’s observation showed that the applied therapy prolonged radiologic response and was achieved with minimal toxicity and an improvement in fatigue [105].

## 6. Another Green Compound as Potential BC Treatment

Another plant that may be used in BC treatment is ginger. Among many ingredients of ginger, [6]-gingerol and [6]-paradol have shown in vitro antitumor activity against different tumoral cell lines [106,107]. An N-butyl-N-(4-hydroxybutyl)-nitrosamine (BBN)-induced animal model of BC confirmed that diet contains ginger extract was associated with a protective effect on the post-initiation stage of urothelial carcinogenesis, reducing the development of proliferative lesions such as hyperplasia and both benign and malignant neoplasia [108]. Moreover, [6]-gingerol can be used as an agent to alleviate the side effects of conventional therapy in cancer patients. A clinical trial confirmed the properties of [6]-gingerol as an antiemetic in patients with solid tumors receiving moderately or strongly emetogenic chemotherapy. Thus, [6]-gingerol may contribute to significant improvement of the appetite and quality of life in cancer patients receiving adjuvant chemotherapy [109].

DPN belongs to pigmented anthocyanidins, which are present in a wide range of fruits and vegetables, such as berries, grapes, sweet potatoes and pigmented cabbages [110]. A previous study suggested that DPN showed potent anti-inflammatory and antioxidant activity and thus can be a promising anticancer agent [111]. Kang et al. (2021) found that DPN induced oxidative stress-mediated apoptotic BC cell death. Moreover, DPN in low concentration (30 and 40 μg/mL) contributed to increased accumulation of BC cells in S and G2/M phase, while in higher concentration (50 and 60 μg/mL), the number of cells arrested at the S and G2/M phase was reduced. This effect is due to the increase in apoptotic cell death at higher concentrations of DPN [112].

**Figure 5:**
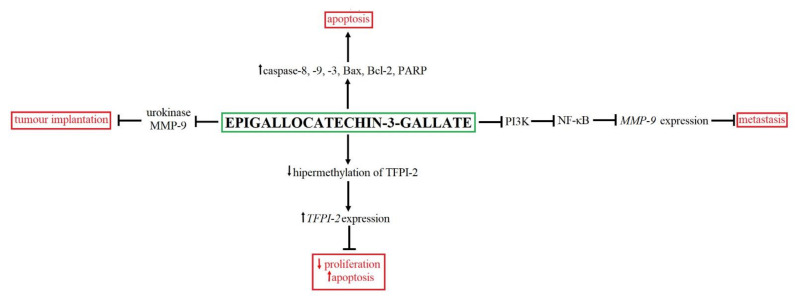
The summary of anticancer effects and mechanism of EGCG action. EGCG may modulate numerous signaling pathways, including cell proliferation [116], metastasis [113,117,118] as well as apoptosis [113,115,116].

EGCG, found in green tea, may effectively inhibit tumors’ formation and development. On the other hand, excessive EGCG uptake may also contribute to the cytotoxicity of normal cells [113]. Among the side effects of excessive consumption, EGCG hepatitis has been observed. Therefore, it was necessary to develop EGCG transport systems to the desired target cells, including BC cells [114]. One such solution is the application of EGCG-adsorbed nanogold particles (pNG), which was administered to C3H/HeN mice subcutaneously implanted with MBT-2 murine bladder tumor cells. The obtained results showed that EGCG-adsorbed pNG suppressed bladder tumor growth through the mitochondria-mediated apoptotic pathway and thus activated the caspase cascade in the absence of the side effects. EGCG-adsorbed, pNG-induced caspase signaling was associated with altering the ratio of Bax/Bcl-2 as well as mitochondrial membrane integrity [113]. Additionally, Hsieh and colleagues (2011) observed that the cell cycle arrest took place in the G0/G1 phase after EGCG-adsorbed pNG treatment. Another in vitro study confirmed that EGCG might induce apoptosis by activation of caspases-8, -9 and -3, Bax, Bcl-2 and PARP [113]. Moreover, an animal study showed that intraperitoneal injection with EGCG reduced by 68.4% the tumor volume [115]. Proliferation inhibition and apoptosis induction by EGCG treatment may also be a consequence of decreased hypermethylation of the tissue factor pathway inhibitor 2 (TFPI-2) gene promoter, leading to an increase of *TFPI-2* expression [116]. Moreover, EGCG may also lower proteolytic activity and the probability of cancer cell implantation. Interestingly, EGCG prevented intravesical tumor implantation more effectively than mitomycin C. Additionally, this study demonstrated that reduced tumor implantation after EGCG therapy might be associated with urokinase and MMP-9 inhibition [117]. An animal study demonstrated that the mice injected with the EGCG–pNG complex were characterized by *VEGF* suppression, indicating the antiangiogenetic potential of EGCG–pNG [113]. On the other hand, the antimetastatic potential of EGCG may be a result of inhibited PI3K/Akt activation by EGCG that may lead to an inactivation of NF-κB and the inhibition of the expression of *MMP-9*. The NF-κB inactivation can result from EGCG-downregulated expression of one of its components, p65 protein, in the nucleus, preventing p65 nuclear translocation by EGCG. Thus, EGCG efficiently and dose-dependently inhibits adhesion, migration and invasion of BC cells [118]. The summary of the antitumor activity of EGCG is presented in Figure 5.

Another interesting natural substance with anticancer properties is gossypol, extracted from cotton plants. In vivo study demonstrated that −(−)gossypol treatment induced apoptosis in both chemosensitive and chemoresistant BC cells. Moreover, gossypol treatments combined with gemcitabine and carboplatin showed synergized effects and induced apoptosis in chemoresistant BC cells by the downregulation of the Bcl-xl and Mcl-1, which was accompanied by the upregulation of the Bim and Puma proteins [119]. However, Mani and colleagues (2019) found that chemoresistant cells are susceptible to treatment with (−)−gossypol, but exhibit an enhanced basal and drug-induced autophagy, which is associated with diminished apoptotic cell death [120].

## 7. Conclusions

Considering the many anticancer properties at the molecular level of natural compounds, such as curcumin, sulforaphane, resveratrol, quercetin, 6-gingerol, delphinidin, epigallocatechin-3-gallate and gossypol and their lack of toxicity, these compounds can serve as additional therapeutic options for BC patients. The anticancer potential of the analyzed green compounds in BC therapy is presented in Table 1.

Although it is clear that described natural compounds have shown excellent anticancer properties, most of the studies were performed in cell-culture and animal models. It is, however, obvious that these anticancer effects of the analyzed compounds should also be confirmed in clinical trials because it cannot be assumed that the results of tests in animal models will hold for humans because of differences in genetics and metabolism profile. Only the complete results of in vitro and in vivo tests will allow for an objective evaluation of “green compounds” in BC therapy.

Moreover, according to clinical studies, some of these compounds, such as RSV, quercetin, are characterized by poor bioavailability and poor stability [32,100,102]. However, this issue may be conquered by new transport strategies based on nanoparticles or encapsulation, which provide more stability and better bioavailability of these compounds in anticancer therapies [91,92,93,103]. Another significant problem is the size of the dose that should be used in the treatment of patients with BC. The doses used in in vitro studies and animal models are significantly lower than the doses that will be used in therapy. The existence of these differences is confirmed by studies on the therapeutic potential of gingerol and quercetin. In the case of studies on cell lines, the maximum concentration of the tested compounds was 160 and 100 µM, respectively, while in the case of clinical studies, the daily dose was 20 mg and 2 g per day, respectively. These differences result from the different course of metabolism of compounds in the in vitro and in vivo systems. In the case of an in vitro system, only a given type of tissue is analyzed, which is represented by the cell line under study. In the case of organisms, we deal with different tissue systems that metabolize a given compound in a different way and with different efficiency. The many metabolizing systems in the organism are also associated with the reduction of the half-life of a given compound, and thus also the time of their actions impact on target cells. Moreover, in vitro tests are so-called an isolated system, which is deprived of the influence of not only the other neighboring tissues but also the influence of environmental factors determining the course of metabolism of the analyzed substances. Therefore, as is well known, the results obtained in vitro cannot be extrapolated to in vivo tests and clinical trials. They can only form the basis for research into the molecular mechanisms of action of compounds [95,96,97,105,106,107,108,109]. The consequence of the high doses of natural compounds that must be taken in order to achieve the anticancer effect is toxicity. Among analyzed compounds, EGCG shows a hepatotoxic effect already during in vitro studies. However, the potential toxicity of natural compounds towards normal cells can be eliminated by developing transport systems that will ensure the release of active substances only at the target site. Concluding, it is necessary to conduct clinical trials that will allow assessing the safety of using natural remedies with anticancer potential and develop effective markers that will predict the effectiveness of the therapy used.

## Figures and Tables

**Table 1:** The anticancer potential of the analyzed green compounds in BC therapy.

Green Compound	Potential Therapeutic Effect	Synergized Therapeutic Effects	The Supposed Problems in Its Clinical Application
Curcumin	- The histone deacetylase (HDAC) inhibitor potential—oral curcumin administration decreased *HDAC* expression without side effects compared to synthetic HDAC inhibitors using clinical practice [16].	- Curcumin combined with BCG therapy may potentiate BCG-induced apoptosis by: ◦ Intensifying activation of caspases 8 and 9 [28]; ◦ Simultaneous increasing the TRAIL receptors upregulation and increased release of TRAIL from peripheral mononuclear neutrophils [28]; ◦ Decreasing NF-κB activity which may lead to downregulated Bcl-2, Bcl-xL and surviving [28].	The dose over 2000 mg per day is associated with neutropenia, diarrhea, abdominal swelling or pain, transient red blood cell echinocyte formation and an increase in the mean red blood cellular volume [37,38,39,40].
	- Apoptosis induction—curcumin treatment led to an increase of p53 level, while down-regulating the antiapoptotic protein Bcl-2; curcumin also causes an increase of Bax expression and caspases-3 and 7 activity as well as reduced surviving level [17,18,19,20].		
	- Antiangiogenic potential—Curcumin may block integrin adhesion receptors and decrease the expression of *VEGF*, *VEGFR1* [21,22,23,24].	- Curcumin combined with gemcitabine may lead to intensification of apoptosis by: ◦ Suppression of COX-2 and VEGF genes [26]; ◦ Upregulating TRAIL and modulating the NF-κB pathway [26].	
	- Regulation of adaptive and innate immunity - curcumin may increase the level of cytotoxic CD8+ T-cells, decrease Tregs and MDSC levels and increase interferon-gamma production; moreover, curcumin may also enhance the cytotoxic effect of NK cells [25,26,27].	- Cisplatin treatment combined with curcumin may: ◦ Potentiate cisplatin-induced apoptosis via ROS-mediated activation of ERK1/2 [33]; ◦ Induce upregulating pro-apoptotic Bax and down-regulate antiapoptotic Bcl-2 and XIAP [33]; ◦ Curcumin may decrease cisplatin-induced nephrotoxicity by reducing serum and renal TNFα and renal MCP-1 concentrations [33].	
	- Interaction with miR-7641, which consequently may cause increased p16 level and induction of the apoptosis [29].		
Sulforaphane (SFN)	- Apoptosis potential by induction of the cytochrome c release from mitochondria, cleavage of caspase-3/9 and PARP, cytoplasmic accumulation of histone-associated DNA fragments and absence of cleavage of caspase-8 [51,53].	No data.	SFN can interfere with successful immunotherapy [61].
	- Induction of cell cycle arrest in S and M phases by the down-regulation of Cdc25C and mitotic spindles disruption [51].		
	- The HDAC and DNMT inhibitors potential [54].		
	- Antiangiogenic potential by the decrease of mRNA expression of *VEGF*, *HIF-1α*, *c-Myc*, *iNOS* and *COX-2* [55].		
	- Disorders to the membrane integrity by suppression of the matrix metalloproteinase-2 and its tissue inhibitor of metalloproteinase-2 transcription [55].		
Resveratrol (RSV)	- Induction of cell cycle arrest in G1 phase by Cyclin D1-CDK4 complex inhibition [75,76].	- RSV and rapamycin combined therapy may inhibit mTOR regulation, reduce cell migration and induce apoptosis [89].	- The antitumuor potential of resveratrol may be effective in miR-21 overexpressing patients [84].
	- Apoptosis potential by p-Akt inhibition and modulation of Bcl-2 family protein activity as well as cytochrome c release from mitochondria and caspase-9 activation [77,78,79,80,81,82].		
	- Inhibition of miR-21, which consequently may cause the reduction of Akt activity, the decrease of *Bcl-2* expression and finally may induce apoptosis [83].	- RSV combination with gemcitabine may lead to an additive cytotoxic effect in the case of gemcitabine-resistant BC cells [32,100].	- RSV use is limited due to its low bioavailability and fast metabolism [32,100].
	- Antiangiogenic potential by the inhibition of MMP-2 and MMP-9 activity as well as reduced expression of the proangiogenic factors, including *VEGF* and *FGF-2* [80,85,86,87].		
quercetin	- Antiproliferation potential by the increase of p-AMPK expression and decrease p-p70s6k and p-4EBP1 expression; moreover, proliferation may be inhibited by hyperpolarisation KCa channels and the reduction of the ecto-5′-nucleotidase/CD73 activity [95,96].	- Quercetin in combined with cyclophosphamide may prolong a radiologic response [105].	Natural quercetin is characterized by low absorption, extensive metabolism and rapid elimination [102].
	- Antiapoptotic potential by the increase of the caspase 3/7 activities and decrease expression of mutP53 protein and survivin [97,98].		
	- Regulation of cell migration through p-Akt and membrane type-1 matrix metalloproteinase (MT1-MMP) pathway regulations [103].		
[6]-gingerol	- Inhibition of the hyperplasia and the neoplasia [108].	No data.	No data.
Delphinidin (DPN)	- ROS-induced apoptosis [111,112].	No data.	No data.
	- Induction of cell cycle arrest in S and G2/M phases [112].		
Galusan 3-epigallokatechiny (EGCG)	- Apoptosis induction by activation of caspases-8, -9 and -3, Bax, Bcl-2, PARP and disorders of mitochondrial membrane integrity [113].	No data.	EGCG may lead to hepatitis [114].
	- Antiproliferation potential by decreased hypermethylation of TFPI-2 gene promoter [116].		
	- Antiangiogenic potential by urokinase and MMP-9 inhibition as well as VEGF suppression [117,118].		

## Data Availability

Not applicable.
